# TRIP - T cell receptor/immunoglobulin profiler

**DOI:** 10.1186/s12859-020-03669-1

**Published:** 2020-09-29

**Authors:** Maria Th. Kotouza, Katerina Gemenetzi, Chrysi Galigalidou, Elisavet Vlachonikola, Nikolaos Pechlivanis, Andreas Agathangelidis, Raphael Sandaltzopoulos, Pericles A. Mitkas, Kostas Stamatopoulos, Anastasia Chatzidimitriou, Fotis E. Psomopoulos, on behalf of the Hellenic Precision Medicine Network in Oncology

**Affiliations:** 1 Department of Electrical and Computer Engineering, Aristotle University of Thessaloniki, Thessaloniki, 54124 Greece; 2 Institute of Applied Biosciences, Centre for Research and Technology Hellas, Thessaloniki, 57001 Greece; 3 Department of Molecular Biology and Genetics, Democritus University of Thrace, Alexandroupolis, 68100 Greece; 4 Dept of Molecular Medicine and Surgery, Karolinska Institutet, Stockholm, Sweden

**Keywords:** Antigen receptor, Software pipeline, R shiny

## Abstract

**Background:**

Antigen receptors are characterized by an extreme diversity of specificities, which poses major computational and analytical challenges, particularly in the era of high-throughput immunoprofiling by next generation sequencing (NGS). The T cell Receptor/Immunoglobulin Profiler (TRIP) tool offers the opportunity for an in-depth analysis based on the processing of the output files of the IMGT/HighV-Quest tool, a standard in NGS immunoprofiling, through a number of interoperable modules. These provide detailed information about antigen receptor gene rearrangements, including variable (V), diversity (D) and joining (J) gene usage, CDR3 amino acid and nucleotide composition and clonality of both T cell receptors (TR) and B cell receptor immunoglobulins (BcR IG), and characteristics of the somatic hypermutation within the BcR IG genes. TRIP is a web application implemented in R shiny.

**Results:**

Two sets of experiments have been performed in order to evaluate the efficiency and performance of the TRIP tool. The first used a number of synthetic datasets, ranging from 250k to 1M sequences, and established the linear response time of the tool (about 6 h for 1M sequences processed through the entire BcR IG data pipeline). The reproducibility of the tool was tested comparing the results produced by the main TRIP workflow with the results from a previous pipeline used on the Galaxy platform. As expected, no significant differences were noted between the two tools; although the preselection process seems to be stricter within the TRIP pipeline, about 0.1% more rearrangements were filtered out, with no impact on the final results.

**Conclusions:**

TRIP is a software framework that provides analytical services on antigen receptor gene sequence data. It is accurate and contains functions for data wrangling, cleaning, analysis and visualization, enabling the user to build a pipeline tailored to their needs. TRIP is publicly available at https://bio.tools/TRIP_-_T-cell_Receptor_Immunoglobulin_Profiler.

## Background

Antigen receptors, namely the B cell receptor immunoglobulin (BcR IG) and the T cell receptor (TR) expressed by the B and T cells, respectively, are characterized by extreme diversity of specificities. This property enables the human immune system to recognize a broad spectrum of exo- and auto-antigens, thus orchestrating a wide range of immune responses fundamental to health (e.g. protection against microbial pathogens or cancerous cells) and disease (e.g. autoimmunity, allergy, lymphoid cancer). Within the last decade, the use of next generation sequencing (NGS) enabled a much deeper and thorough study of both BcR IG and TR gene repertoires through the generation of an unprecedented amount of sequence data offering a profound impact on our understanding of various clinical and research settings.

On one hand, immune profiling using NGS methodologies is gaining popularity in the context of cancer and autoimmunity through monitoring minimal residual disease (MRD) and characterizing the complexity of the immune repertoires, respectively. On the other hand, IG/TR NGS can also provide valuable information regarding normal processes and mechanisms, such as B and T cell development, inflammation and the aging of the immune system. In contrast to the vast majority of (human) genes, the analysis of BcR IG/TR rearrangement sequences cannot be based on simple comparison with a reference genome. This is due to the fact that: (1) BcR IG/TR receptor variable domains are created through the combination of 2 (V and J) or 3 (V, D, and J) types of genes, and, (2) random nucleotides are deleted and/or inserted at the junctions between these genes, thus resulting in a high level of sequence diversity.

NGS methodology has been applied in the field of immunogenetics in three main areas: (i) clonality assessment, (ii) detection of minimal residual disease (MRD) and (iii) repertoire analysis of BcR IG and TR gene sequences [1–3].

The implementation of NGS technologies, hence the capacity to generate massive amounts of data, underlies the requirement for powerful computational and analytical tools while also raising challenges in data sharing, archiving, and aggregation. Therefore, a series of different pipelines have been developed to facilitate both data annotation and meta-analysis [4–7].

In this context, the international ImMunoGeneTics (IMGT) [8] information system is the most widely used repository and curation site for BcR IG and TR gene sequence information. IMGT is also able to computate clonotypes defined as unique combinations of variable (V), diversity (D), joining (J) genes and complementarity-determining region 3 (CDR3) amino acid (AA) in-frame junction [9]. Each "IMGT clonotype (AA)" is characterized by a selected unique representative sequence. The "IMGT/StatClonotype" tool can evaluate and visualize statistical significance of pairwise comparisons of IMGT clonotype (AA) diversity or expression per gene of a given BcR IG or TR dataset, which should not exceed 1,000,000 sequences. This tool is incorporated in a downloadable R package with user-friendly interface [10, 11].

Another software for comprehensive adaptive immune profiling is MiXCR [12]. MiXCR handles both paired- and single-end reads, depending on the utilized sequencing chemistry, assesses their quality and applies a heuristic multilayer clustering for error correction. It also aligns the reads with a built-in library of reference germline (D)J and constant (C) gene sequences for humans and mice based on the corresponding loci from GenBank. MiXCR computates clonotypes by assembling identical and homologous reads and is also able to rescue low-quality reads by mapping them to previously assembled high-quality clonotypes. The software is able to analyze full- and partial-length data.

The Vidjil platform [13] is an open-source application for the analysis of high-throughput sequencing reads of BcR IG and TR gene rearrangements. The algorithm performs the processing and annotation of one or several samples and displays the results in an interactive user-friendly interface. The data can be stored and analyzed by several complementary software, including an annotation step through IMGT. As input, the user needs to provide a raw sequence file. The algorithm identifies clonotypes by default based on a so-called "window" of 50 bp nucleotides within the V(D)J junction and proceeds with the alignment of the sequences. The window size can be changed by the user. The algorithm then assesses the clonality of the samples, displays the most frequent clonotypes of each sample in lists and visualizes the results. Vidjil has also been designed to quantify MRD detection during patient follow-up.

Another software toolbox for high throughput immune receptor profiling is available on the Galaxy platform [14], namely the Immune Receptor Profiler (IRProfiler). The selection of Galaxy as the hosting platform of IRProfiler ensures the usability and modularity of IRProfiler and provides powerful means for its distribution. IRProfiler uses the "Summary" file from IMGT/HighV-Quest tool as input and can use 5 alternative clonotype definitions, performs data filtering, clonotype computation and gene usage for the V and J genes of both BcR IG and TR. Moreover, IRProfiler is able to detect shared and exclusive clonotypes among different repertoires and datasets and compare V and J gene repertoires among different samples. The pipeline of IRProfiler has been employed by a number of publications for TR repertoire analysis in different pathological conditions [14].

In this paper, we present the T-cell Receptor / Immunoglobulin Profiler (TRIP) tool, a software framework that provides analytical services on BcR IG and TR gene sequence data. TRIP offers the opportunity for an in-depth analysis based on the processing of the output files of the IMGT/HighV-Quest tool, and was developed to address a variety of scientific issues in NGS IG/TR data analysis ranging from data curation and filtering to the characterization of complex features and processes. Datasets from many different patients can be processed together and the results can be displayed either together as a merged output or separately. In detail, TRIP performs clonotype computation, the definition of which is specified according to user needs. Highly similar clonotypes, i.e. BcR IG or TR clonotypes using the same V gene and differing in a few amino acids within the CDR3 of a particular length, can also be computed, while shared clonotypes between different datasets (either samples or individuals) can be identified. The V, D and J gene repertoires can be extracted and the relations between different characteristics of the gene rearrangements can be evaluated, e.g. V gene usage and CDR3 length. Molecular features of the receptors can be assessed in a comprehensive way, such as CDR3 predicted isoelectric point value and length distribution and (only for BcR IG genes) somatic hypermutation (SHM) status and characteristics. Specifically regarding the latter, TRIP has the option to align rearranged IGHV genes against the germline gene and allele (from IMGT) and assess the frequency and characteristics of the observed mutations based on their physicochemical properties [15]. The results can be displayed in a web interface and all outputs can be downloaded in a text (.txt) format.

A key advantage of TRIP is that it offers the opportunity for comprehensive data analysis for both antigen receptors through a unified, reproducible and user-friendly interface. In addition, it supports a wider range of user experience; from a fully interactive graphical interface, to a fully customizable and versatile commandline tool that can be incorporated in further workflows.

TRIP has already been used in studies of hematologic malignancies assessing the immune repertoire in different contexts like chronic lymphocytic leukemia (CLL) and multiple myeloma (MM) [16–18], supporting theories for antigen involvement into disease pathophysiology. Moreover, in recent studies of the team TRIP was implemented for the characterization of the intraclonal temporal dynamics leading to clonal drift in CLL, as well as the subclonal ’architecture’ essentially arising from intraclonal diversification of the BcR IG genes in the context of ongoing SHM, alluding to interactions with disease-specific antigens [19, 20].

Additionally, the implementation of TRIP for NGS TR data analysis in the context of HIV supported an antigen-driven, HIV-specific immune process in the development of non-neoplastic reactive lymphadenopathy, through the detection of clonotypes with established reactivity against certain HIV protein epitopes [20]. Similar studies focusing on cell lines enriched for T cells reactive against viral infections (e.g. by EBV and/or CMV and/or BKV), a major cause of morbidity and mortality after allogeneic hematopoietic stem cell transplantation, provided insights into the TR repertoire of ex vivo- or endogenously-generated virus-specific T cells [21].

## Implementation

In order to ensure consistency in the description of each functionality offered by the tool, the following list of definitions will be used:

AA Junction: The AA junction refers to the unique V-(D)-J gene rearrangement leading to the AA sequence between the conserved anchors (e.g. C104 and W118 for the heavy chain of the BcR IG).

**Clonotype**: From a biological perspective, is a unique nucleotide sequence that arises during the gene rearrangement process for BcR IG or TR. There are several definitions that can be used according to the needs of each study, i.e. a unique combination of V-(D)-J gene and allele and/or the CDR3 at amino acid or nucleotide level.

**Highly similar clonotypes**: It refers to clonotypes with the same CDR3 length that differ in the AA composition in few particular positions.

**Shared clonotypes**: The identification of common clonotypes between different datasets and their relative frequencies in every dataset.

**Convergent evolution**: This term is used to describe the phenomenon of different nucleotide sequences degenerately encoding the same CDR3 amino acid sequence [22–25].

Moreover, it should be noted that the tool functions have been explicitly designed to work at both the nucleotide and the amino acid level, depending solely on the requested task.

**Fig. 1:**
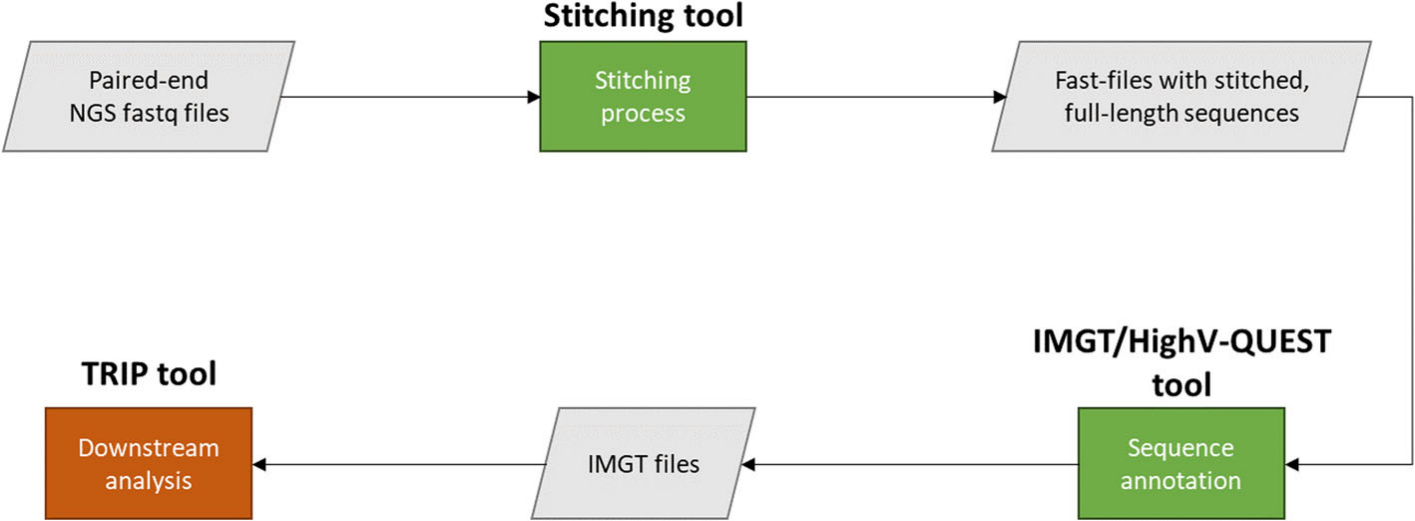
Overall workflow of the NGS data analysis; the TRIP tool is used for the downstream analysis of the NGS data, after the stitching process and the sequence annotation offered by IMGT/High-VQuest

A typical NGS data analysis workflow is depicted on Fig. 1. BcR IG and TR NGS paired-end protocols produce anti-parallel reads, Read 1 (R1) and Read 2 (R2) providing with both forward and reverse reads for each amplified BcR IG/TR gene sequence. The first step of the analysis is to filter R1 and R2 raw sequences according to a set of quality rules. Subsequently, the paired-end reads are combined based on an overlapping region and the stitching process leads to the generation of the full-length sequences, which undergo quality filtering again. The output of the algorithm consists of 10 files including the FASTA files with stitched, full-length sequences, statistics and reports about the process. The FASTA file of the stitching algorithm serves as input for the IMGT/HighV-QUEST Tool, which provides the most up-to-date reference dataset for human BcR IG and TR genes and offers characterization of clonotypes for clonal diversity. As output, the tool produces an archive file, which comprises 12 different files. This output is used as input to the TRIP tool for meta-data analysis.

TRIP can be used in four ways: a) as a standalone graphical tool running locally on your computer, b) as a web application hosted on a server, e.g. the Amazon cloud server, c) as a docker container, and d) as a script-based tool. The graphical environment of TRIP is implemented in R Shiny, an R package that can be used to build interactive web applications straight from R, allowing users to directly interact with the data, the analysis and the results. So, in the first case, R, R Studio, the R Shiny package and all the other R packages that TRIP uses are installed. In the second case, a Shiny App can be defined as a web page (User Interface, UI) connected to a computer running a live R session (Server). The users are able to access the web page through any device (no dependencies needed) and select personalized parameters via the UI. The selected parameters are passed to Server, where calculations are performed, and the UI’s display is updated according to them. In the third case, the docker container of TRIP can run in cloud infrastructures as well as in any machine running the Docker daemon, without further installations. The docker image of TRIP is publicly available through DockerHub, whereas more information about Docker can be found in [26]. Finally, in the last case, TRIP can run as an R script-based tool, where all parameters can be selected through the command line. More information about this tool are available in the project’s github repository.

To create an R Shiny application, two predefined R scripts need to be located into the same directory. The first one implements the User Interface (ui.R) by controlling the layout of the page using html commands and other nested R functions, and handling the input parameters inserted by the users. The second script implements the Server (server.R) and contains essential commands and instructions on how to build the application and process the data. Apart from these scripts, TRIP also includes a helper script (helpers.R), which contains all the created functions needed for the whole analysis.

The main R package that an R Shiny application needs is the shiny package. To customize the UI, additional CSS and Javascript libraries can be used within the application, such as shinyjs and shinyBS. In order to handle big data, data structures that use indexing and keys are used to save data and make the look-up process quicker, as well as libraries that contain functions with an option of parallelize (i.e dplyr, data.table, tidyr). The results of the analysis are presented using plotting packages, such as plotly and plot3D.

**Fig. 2:**
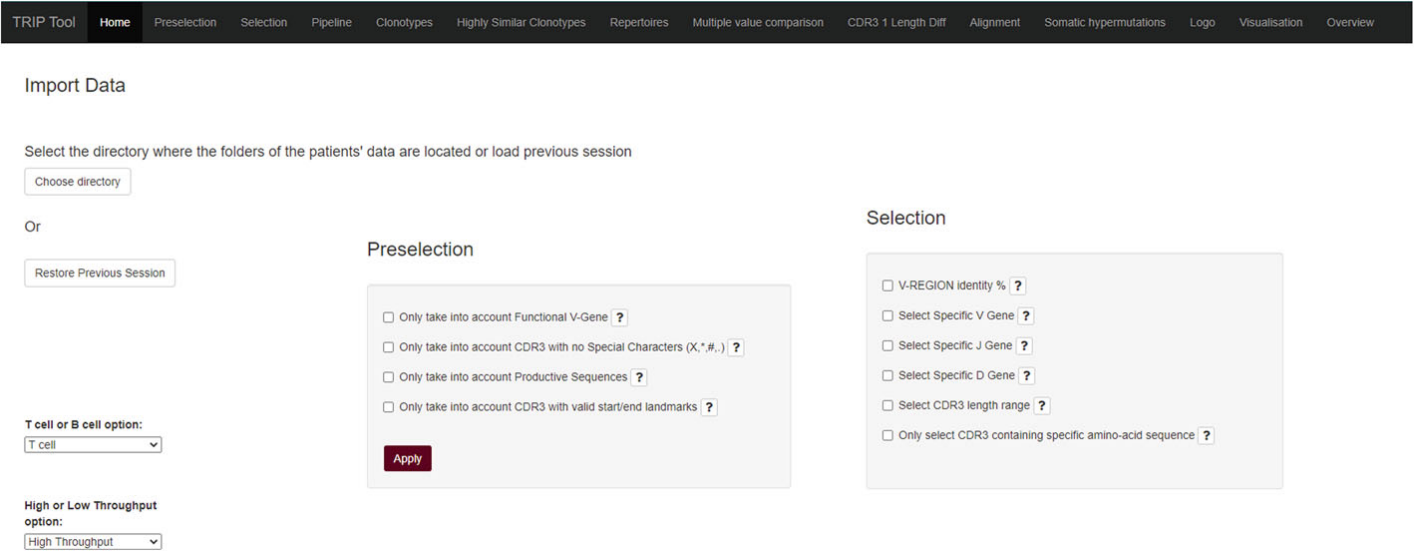
The Home page of TRIP tool: Users can import data by selecting the directory in which the data is stored. Previous sessions can also be loaded ("Restore Previous Sessions" button). There are 2 options regarding the receptor analyzed (BcR IG and TR) as well as 2 options based on the type of available data (high- or low-throughput). In the next step, filters are applied to the chosen data for curation (Preselection) and filtering (Selection)

**Fig. 3:**
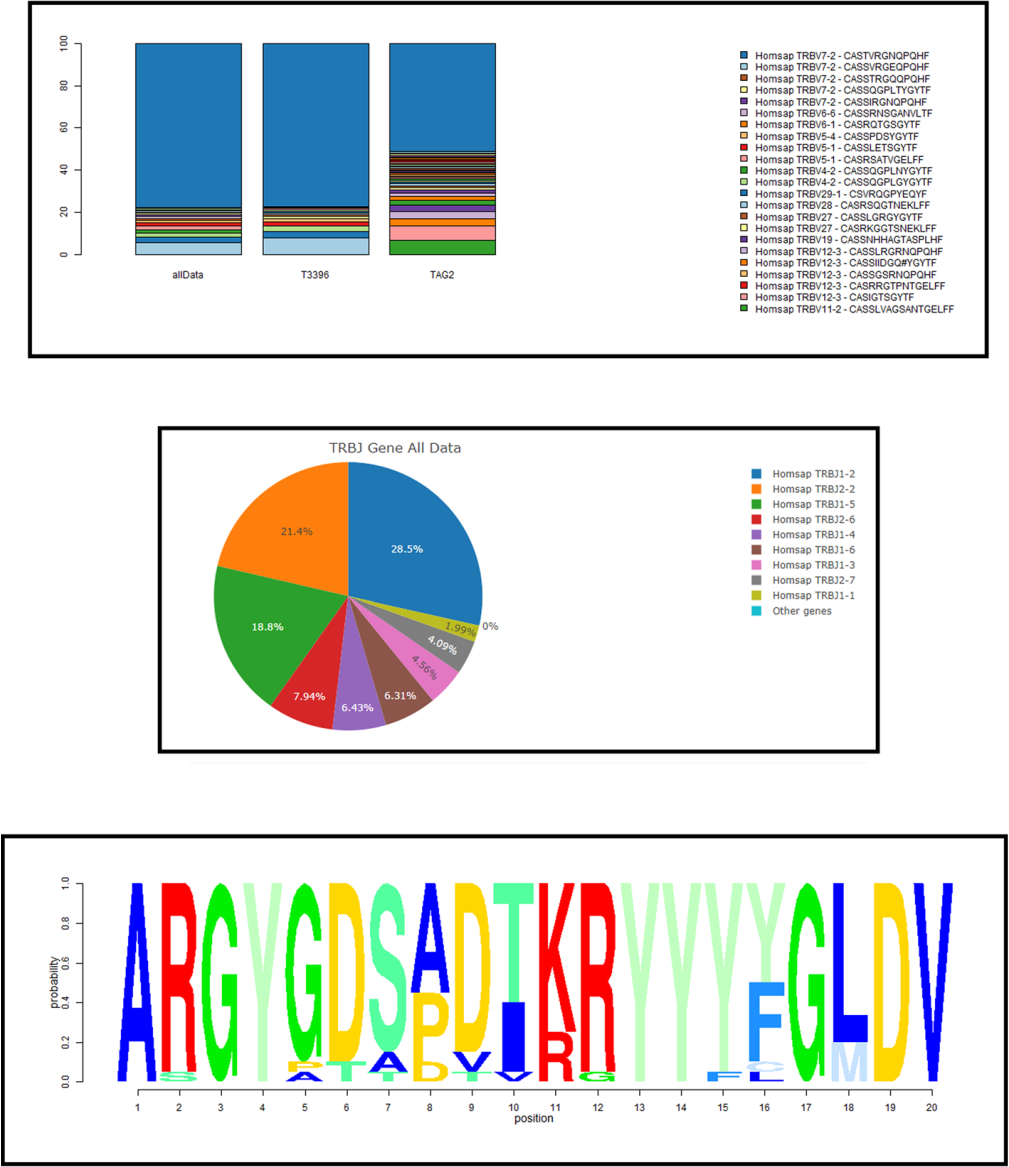
Example visualization plots from TRIP: (top) Clonotypes barplot for each sample and the total dataset analyzed, (middle) Repertoires pie chart and (bottom) Logo graph of the CDR3

**Fig. 4:**
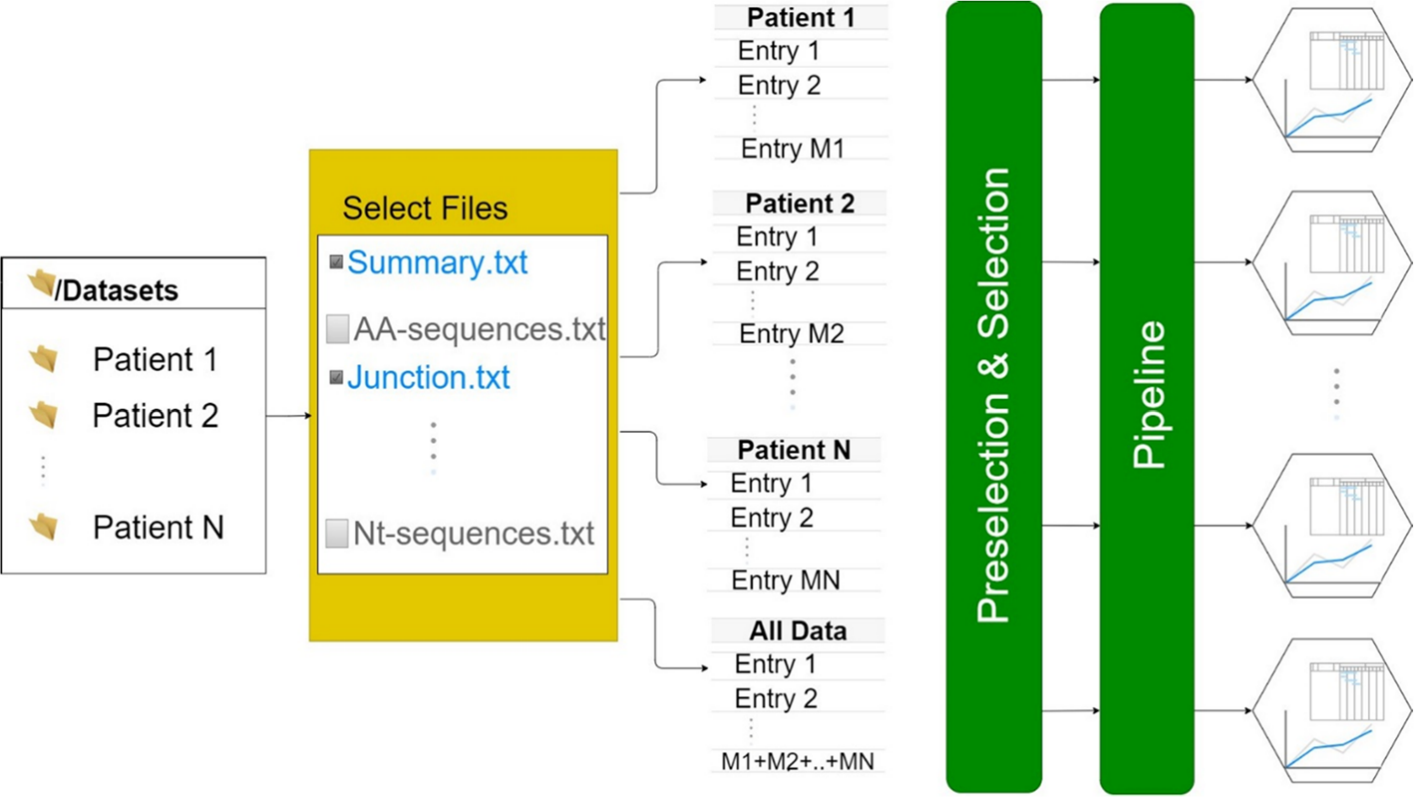
The workflow of the TRIP tool. Overall schema of the TRIP tool usage. Initially, the user selects the desired patients’ datasets (i.e. IMGT/HighV-Quest files), performs data cleaning (preselection) and filtering (selection), and executes the preferred pipeline

**Fig. 5:**
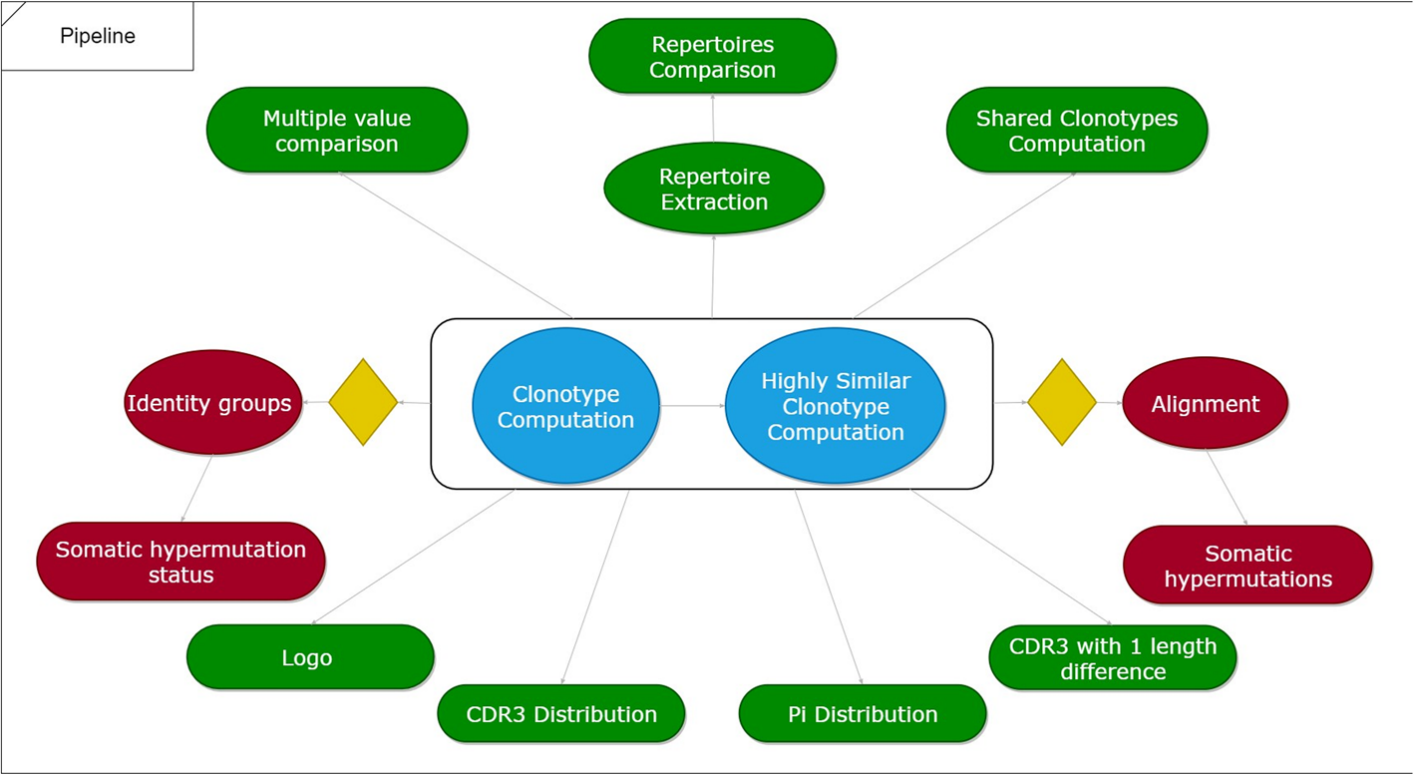
Overview of TRIP tool’s pipeline: The two main functionalities (Clonotype and Highly Similar clonotype computation – represented with a blue) as well as the dependencies between the rest of the pipeline’s functions are illustrated above. Clonotype computation is the only step that is required for the pipeline to run, while every other procedure is optional. Green colored functions can be used for both T or B cells, while red colored function can be run only for B cells

The user interface of TRIP is organized in 12 major tabs (Fig. 2), including the Home tab, the Preselection and Selection tabs, the Pipeline tab, one tab for each one of the steps of the pipeline with the corresponding results, and the Visualization tab. The default pipeline of TRIP depends on the antigen receptor type (BcR IG or TR) and includes the following procedures: clonotype computation, highly similar clonotype computation, shared clonotype computation, repertoire extraction, repertoire comparison, multiple variable comparison, sequence alignment, SHMs and amino acid position-based frequency estimation. Some examples of the produced visualizations are available in Fig. 3. The user is able to form a personalized pipeline by selecting the processes that fit their needs and taking into account the dependencies between the processes. The whole workflow and the pipeline processes that are available, including the aforementioned dependencies between them, are presented on Figs. 4 and 5, accordingly. At the end of the process, the whole session, i.e. the user-selected parameters and the function outputs, can be bookmarked, in order for the user to be able to restore it any time.

### Load data and initialization

The first step of the process is to upload the input data, which comprises the output files of the IMGT/HighV-Quest tool [27]. By design, TRIP currently supports as input the output files produced by the IMGT/HighV-Quest tool. This is due to the fact that IMGT outputs are used as a global standard format for the field of immunogenetics. However, IMGT provides an option for downloading the output files in AIRR format (Adaptive Immune Receptor Repertoire Standards - https://docs.airr-community.org/) which provide a more concrete schema for describing, analyzing, storing and sharing immunogenetic data. Following this, and taking into account that similar tools in the field support both IMGT and AIRR formats (e.g.Vidjil – http://www.vidjil.org/), future extension of TRIP includes supporting outputs coming from both IMGT or AIRR tool.

Data from a given sample are organized into a folder containing 10 individual files in text (.txt) format. Users are able to choose to upload only some of these files depending on the type of the downstream analysis. IMGT/HighV-Quest has a submission threshold of 500,000 sequences. If a single sample has more sequences, the data is split into batches of 500,000 sequences through the stitching algorithm. Hence, multiple folders for these given samples will be generated. These folders should be named with the same identifier but a different extension in the form of "-0", "-1" etc. in order to be handled as one sample by TRIP.

Immediately after the upload, input data is automatically checked for the presence of data columns with a different/unknown title. In case such columns are identified, users are asked to replace the names of these columns with the appropriate ones, ensuring that TRIP operation will not be affected by IMGT changes in updated versions. Data columns that will not be used in the downstream analysis as well as duplicated columns are removed at the very beginning of the process to reduce the overall complexity. After this, one table for each sample and a merged table (All Data table) that combines all samples’ data are created.

There are 2 global parameters at the Home tab regarding the antigen receptor type (BcR IG and TR) as well as the type of the data to be analyzed, i.e. high-throughput (NGS) or low-throughput (Sanger sequencing) data. Concerning the latter, the main difference comes to the preprocessing steps, i.e. the Preselection and Selection steps. In the case of high- throughput data, all filters are applied consequentially (i.e. if a sequence fails in more than one selection criteria, only the first unsatisfied criterion will be reported), whereas for low-throughput data all criteria are applied at the same time and only those rows that fail in all criteria are filtered out from the analysis.

### Data preparation

After uploading data and initializing the global parameters, data Preselection (curation) and Selection (filtering) are applied, according to the user’s preferences.

#### Preselection

The Preselection process comprises the following criteria:

1. *Select type of sequences that will be taken into account*: The user has the choice of including only productive sequences (without pseudogenes and/or stop codons and/or frameshifts), only unproductive sequences, or all sequences in the next steps of the analysis.
2. *Only take into account CDR3 with valid start/end landmarks*: Start/end CDR3 landmarks (anchors) can be customized by the user based on the type of data i.e. BcR IG (heavy/light chain) or TR. More than one valid landmark can be requested. Sequences with landmarks other than the chosen ones are excluded from the analysis.
3. Only take into account CDR3 with no Special Characters (X,*): Only sequences without ambiguities (i.e. characters other than those of the 20 amino acids) are included in the analysis.

The results of the Preselection process are presented in the Preselection tab. The output consists of 4 different tables: (i) a summary table with the numbers of both the included and excluded sequences for each different criterion, (ii) the raw data, (iii) the data that meet the Preselection criteria and are provided as input to the selection process and (iv) the excluded raw data, including information about the unsatisfied criterion.

#### Selection

The sequences that passed through the Preselection process are used as input for the data Selection (filtering) process, which comprises 6 different filters:

1. *V-REGION identity %*: Sequences with identity percent to germline that do not fall in the range set by the user are excluded from the analysis.
2. *Select Specific V, J, D Gene*: The user can select for rearrangement sequences of one or more particular V, D, J genes or gene alleles, respectively.
3. *Select CDR3 length range*: Only sequences with CDR3 length within the range set are included in the analysis.
4. *Only select CDR3 containing specific amino-acid sequence*: Sequences with the specific CDR3 amino acid motif provided by the user are included in the analysis.

The results of the Selection (filtering) process are presented in the Selection tab, using the same four tables and layout as those provided at the Preselection tab.

### Pipeline

After the preselection and selection processes, the main pipeline that the user can select consists of the clonotype computation, highly similar clonotype computation, shared clonotype computation, repertoire extraction, repertoire comparison, multiple variable comparison, sequence alignment (only for BcR IG), SHM (only for BcR IG) and amino acid position-based frequency estimation.

#### Clonotype computation

The first and necessary step of the pipeline for both BcR IG and TR is the clonotype computation. The clonotype definition is specified by the user. There are 10 different options for the definition: clonotypes can be defined using a gene (V gene/gene allele, J gene/gene allele) in combination with the CDR3 at nucleotide or amino acid level (AA CDR3 or Nt CDR3) or using only the AA CDR3 or Nt CDR3.

**Fig. 6:**
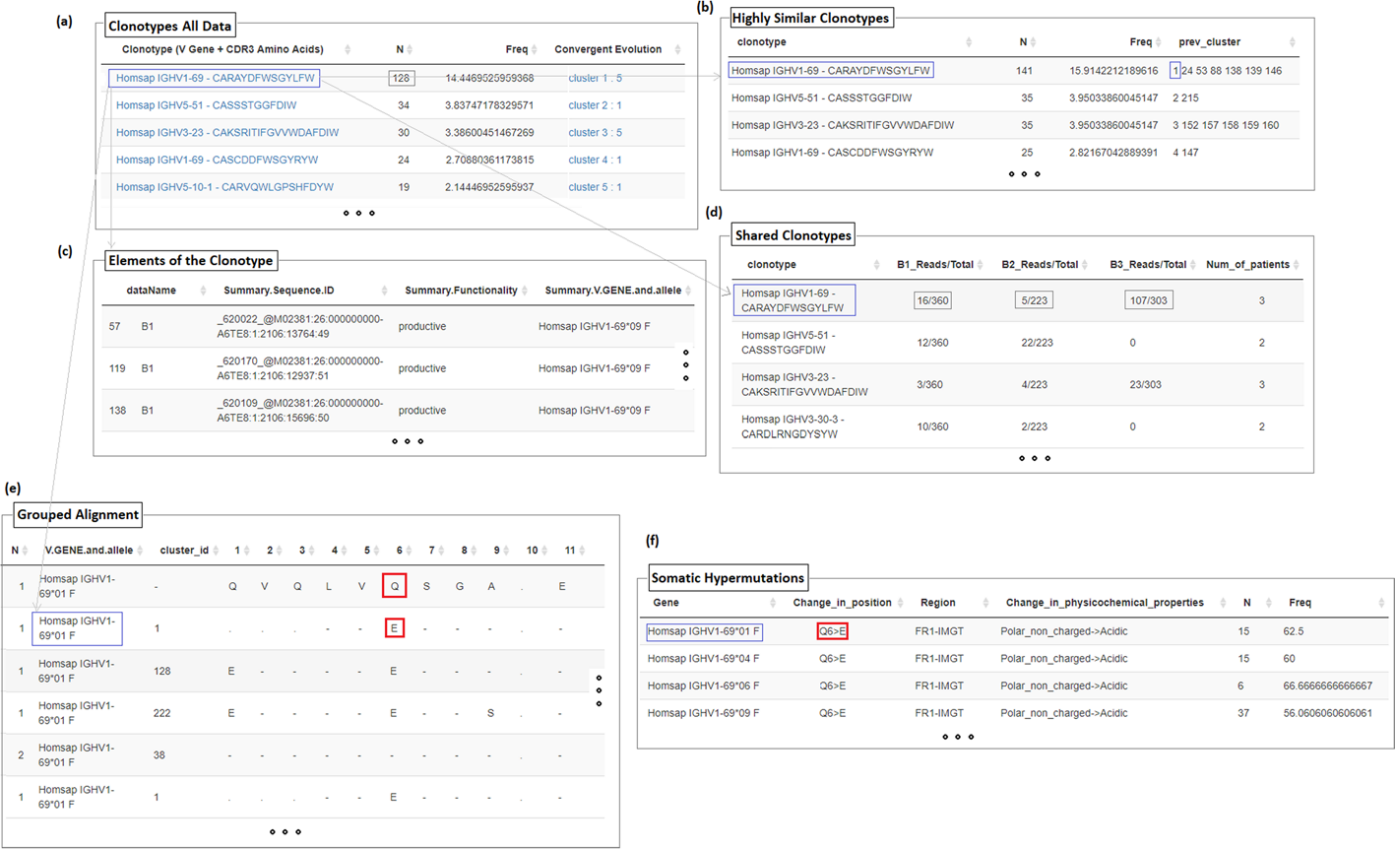
Examples of output tables provided by TRIP. **a** Clonotypes of all datasets. Each clonotype is presented according to the definition chosen by the user followed by its absolute count of reads, its relative frequency and the convergent evolution, i.e. the different nucleotide sequences encoding the amino acid sequence of each clonotype. **b** Highly Similar Clonotypes. Clonotypes of the same CDR3 length and differences in the amino acid composition in few positions are merged. Each clonotype is presented followed by the newly-calculated absolute count of reads, its relative frequency and a list of the cluster ids merged. **c** Elements of the clonotype. Each clonotype presented in the first column of Fig. 1a is also a link that provides a table with all relevant immunogenetic data for that particular clonotype. **d** Shared Clonotypes. When multiple datasets are analyzed simultaneously, some clonotypes may co-occur in more than one datasets. The clonotypes that were present in >= 2 samples are presented in this table followed by the number of reads assigned to each clonotype in every dataset. The last column of the table is about the number of datasets/samples that shared each clonotype. **e** Grouped Alignment. This output table is provided after the alignment and the grouping of the identical sequences at amino acid level based on the reference sequences and each row represents a unique amino acid sequence. It includes the number of reads that are identical, the IGHV gene and allele used as reference, the cluster id (clonotype) the sequence corresponds to (the reference sequence is characterized with "-") and the positions of the BcR IG molecule. The amino acids that correspond to the germline are replaced with "-", while the differences remain. **f** Somatic Hypermutations. This output table is computed based on the alignment table (Fig. 6e) and includes: (i) the gene and allele used, (ii) the actual mutation stating the amino acid of the reference sequence and the new one including the position of the change, (iii) the region where the change occurs based on IMGT, (iv) the type of the mutation with regards to the physicochemical properties, (v) the number of sequences carrying the particular change and (vi) the relative frequency of every mutation

To form clonotypes, the raw data are grouped by the selected pair gene-CDR3 or the selected CDR3 attribute. The relative frequency with which each one of the clonotypes appears into the raw data is computed based on the total raw reads of each sample and the results are then sorted in descending order regarding the frequencies, so that the most frequent-important clonotypes appear at the top of the table. After computing this grouped table (Fig. 6a) and assuming that M clonotypes were uncovered, M sub-tables (*C*_*i*_) are created containing the raw data that correspond to each specific clonotype (Fig. 6c). In the case that AA CDR3 has been selected in the clonotype definition, the number of different CDR3 sequences at nucleotide level that form each clonotype, named as convergent evolution, is computed utilizing the information of the subtables.

#### Highly similar (HS) clonotypes computation

After clonotype computation, the user is able to merge clonotypes that are highly similar (Fig. 6b). On TRIP the threshold used to determine what is considered similar is set by the user and concerns the amino acids themselves and not their physicochemical properties. The user can set the number of mismatches allowed for each CDR3 AA length found in the dataset and a clonotype frequency threshold (*CFTh*), that defines the minimum frequency value that a clonotype can have in order to be considered as major. The process applied for each identical CDR3 length *L* contained in the dataset is described in the following steps.

1. Create subset-*L* which contains clonotypes that are characterized by CDR3 sequences of AA length *L*.
2. Find the clonotype of subset-*L* with the highest frequency. If the frequency is above *CFTh*, this clonotype is considered as the major clonotype of subset-*L*. In this case, go to step 3, otherwise, no clonotypes of length-*L* can be merged, so they remain as they are and the process terminates for length *L*.
3. Find those clonotypes of subset-*L* that have less mismatches with the major one than those allowed by the user for the specific length and assign them to the major group.
4. Create a new subset-*L* with the remaining clonotypes i.e. those that have not been assigned to another group and repeat steps 2 and 3.

The whole process can be performed with or without taking into account the rearranged V-gene. Except for the highly similar clonotypes table which has a similar structure as the clonotype table, one table for each CDR3 length containing information regarding the highly similar clonotype grouping is also provided.

#### Repertoire extraction

The repertoire extraction process follows the clonotype computation process. Six different repertoires can be extracted, i.e. V, J or D gene and/or gene allele. To compute repertoires based on the same gene/gene allele that is used for the clonotype definition, the process is quite simple. The only thing that needs to be done is to group the clonotype table by the gene attribute, compute the relative frequency with which each gene appears in the clonotype table based on the total number of clonotypes identified and sort the results in descending frequency order. In any other case, assumming that the user has selected gene *G*, the repertoires are extracted using the following steps:

1. For each sub-table *C*_*i*_ find the most frequent gene/gene allele of *G*, *G*_*i*_, as follows:
  i Group *C*_*i*_ by column *G*.
  ii Find the frequency of occurrence for each value of *G*.
  iii Sort by frequency in descending order.
  iv Select the first element of column *G* of the grouped table.
2. Gather all *G*_*i*_ values and create a data frame *GDF* with one column.
3. Group table *GDF* by *G*, compute the corresponding frequencies and sort the table to get the repertoire table.

This step of the pipeline can be implemented either on the initial clonotypes or the highly similar clonotypes, in the case that the latter has been computed.

#### Multiple value comparison

Another grouping process which is based on the clonotype table is the Multiple Value comparison process. Here, the user is able to select two attributes (*A*_1_, *A*_2_) and find the frequency that each unique combination of the attributes occurs in the clonotype table. Many different combinations can be selected by the user, including Gene, CDR3-IMGT AA length, the isoelectrical point (pI) etc. To compute the Multiple Value comparison table, the process that is followed is similar to the one followed to extract repertoires using a different gene/gene allele than the one used for clonotype definition, but this time the number of attributes is two:

1. For each sub-table *C*_*i*_ find the most frequent value of *A*_1_, *A*_1*i*_: i) group *C*_*i*_ by *A*_1_, ii) find the frequency of occurrence for each value of *A*_1_, iii) sort by frequency in descending order, and iv) select the first element of column *A*_1_ of the grouped table.
2. Repeat step 1 for *A*_2_ to obtain *A*_2*i*_.
3. Gather all *A*_1*i*_ and *A*_2*i*_ values and create a data frame *ADF* with two columns.
4. Group table *ADF* by the combination *A*_1_ and *A*_2_, compute the corresponding frequencies and sort the table to get the multiple value comparison table.

#### Shared clonotypes computation

After clonotype computation, a table of the shared clonotypes among the different samples that are loaded can be computed. This table is computed by extracting those clonotypes that tend to co-occur in more than one datasets/samples of the analysis (Fig. 6d). This step of the pipeline can be implemented on both the initial clonotypes and the highly similar clonotypes, in the case that the latter have been computed.

#### Repertoire comparison

After repertoire extraction, a comparative repertoire analysis can be performed, when more than one samples are analyzed, using the same approach as the one used for shared clonotypes computation. This step of the pipeline can be selected for both the initial repertoires and the highly similar repertoires, in the case that the latter have been computed.

#### Detect CDR3 sequences with 1 length difference

By selecting this process, groups of similar CDR3 sequences are created. In this case, two CDR3 sequences are called similar when their lengths differ by 1 AA and if one copies the AA of the specific position P to the position *P*+1 of the sorter sequence, the same sequence occurs. Assuming that the CDR3 length of the longest sequence is *L* and the position where the difference can appear is *P*, the process that is applied to each unique gene of the dataset is summarized in the following steps

1. Filter in row data which contain CDR3 sequences with length=*L* or *L*-1.
2. For the CDR3 with length=*L*-1:
  i Make a right sift of the amino acids that belong to the positions (*P*+1):(*L*-1).
  ii Copy the amino acid of the position *P* to the position *P*+1.
3. Group by CDR3, count and summarize.
4. Return the groups with more than one elements

#### Alignment

An alignment table can be created for the selected V region at both Nt and AA level. This can be done by applying the following steps to each unique gene of the dataset:

1. Find the germline of the gene from the corresponding file.
2. Compare the selected region with the germline and replace with "-" the positions of the sequences that correspond to the germline.

A grouped alignment table is created as well by further grouping together the exact same sequences of each particular cluster id (Fig. 6e).

#### Somatic hypermutations

After the alignment process, SHMs can be extracted based on the grouped alignment table. The output table includes: (i) the mutation type, (ii) the position of the change, (ii) the region where the change occurs (based on IMGT unique numbering), (iii) the number of sequences carrying each change and (iv) the frequency of the change for every gene or allele based on the grouped alignment table regardless the clonotype (Fig. 6f). There is the possibility to analyze only a number of clonotypes (top N clonotypes) or even some clonotypes separately.

#### Logo creation

At this step, a logo can be created for the V region or the CDR3. To create a logo, the corresponding length of the sequences must be provided by the user. Moreover, a frequency table needs be computed first, by counting the appearance of the 20 different amino acids at each position of the sequence. There is also the option to include only those sequences at the plot that correspond to the top *N* clonotypes.

### Visualization

In the Visualization tab different types of charts (scatter plots, bar plots, 3D plots, surfaces, histograms etc.) are available for the visualization of the analysis results. Clonotypes are presented as bars and the user can select the frequency above which the clonotypes will be presented. Convergent evolution can also be visualized with more than one chart type options. The computed repertoires are presented as pie-charts and the user can again select the minimum frequency of the gene/allele that will be presented. All computed tables can be downloaded in text format, whereas the plots and the graphics can be downloaded in png format.

## Results and discussion

Two sets of experiments have been performed in order to evaluate the efficiency and the performance of the TRIP tool. Therefore, this subsection is divided into two parts: **(a)** the performance experiments on different sizes of input data regarding memory usage and computational time discussed in Performance testing subsection, and **(b)** the application of the TRIP pipeline in a group of patients presented in Experimental results on a group of patients subsection.

### Performance testing

TRIP was developed as an R Shiny application. Even though R is considered as a slower language, as it makes certain sacrifices for convenience at the expense of optimal speed, a lot of methods can be applied in the coding style to produce efficient R code. What is more, R gives the opportunity to identify bottlenecks and code it directly in C++ via the Rcpp package. A lot of packages, such as tidyverse, take advantage of this, which is why in some cases they may be faster than some base implementations. Based on this, optimization of the system’s time was achieved by vectorizing all data structures in the source code and replacing, when possible, for loops with apply functions. In addition, data processing was performed with the use of the tidyverse package’s functionalities. In this way TRIP allows the user to repetitively perform an analysis on multiple chunks of data coming from multiple patients. However, it should be stated that part of the future work on the tool is to support parallel processing of the input data.

The first set of experiments focused on evaluating the performance of the TRIP tool, in terms of memory usage and computational time. During this set of experiments, we used four different artificial datasets that were created after merging a number of BcR IG gene sequence datasets with similar characteristics, in order to create realistic synthetic datasets. Each one of the four synthetic datasets had 250,000 rows. The datasets are available to the readers (see Availability of data and materials section). The whole pipeline for antigen receptor type BcR IG was applied to all four datasets at the same time, using the initial datasets and subsets of them of different sizes, summing up to 250,000, 500,000, 750,000 and 1,000,000 rows. The experiments were executed on a server that had the following specifications: Ubuntu 18.04.3 LTS (kernel 4.15.0.58-generic), 2 x Intel Xeon X5650 @ 2.67 GHz and 118 GB RAM.

**Fig. 7:**
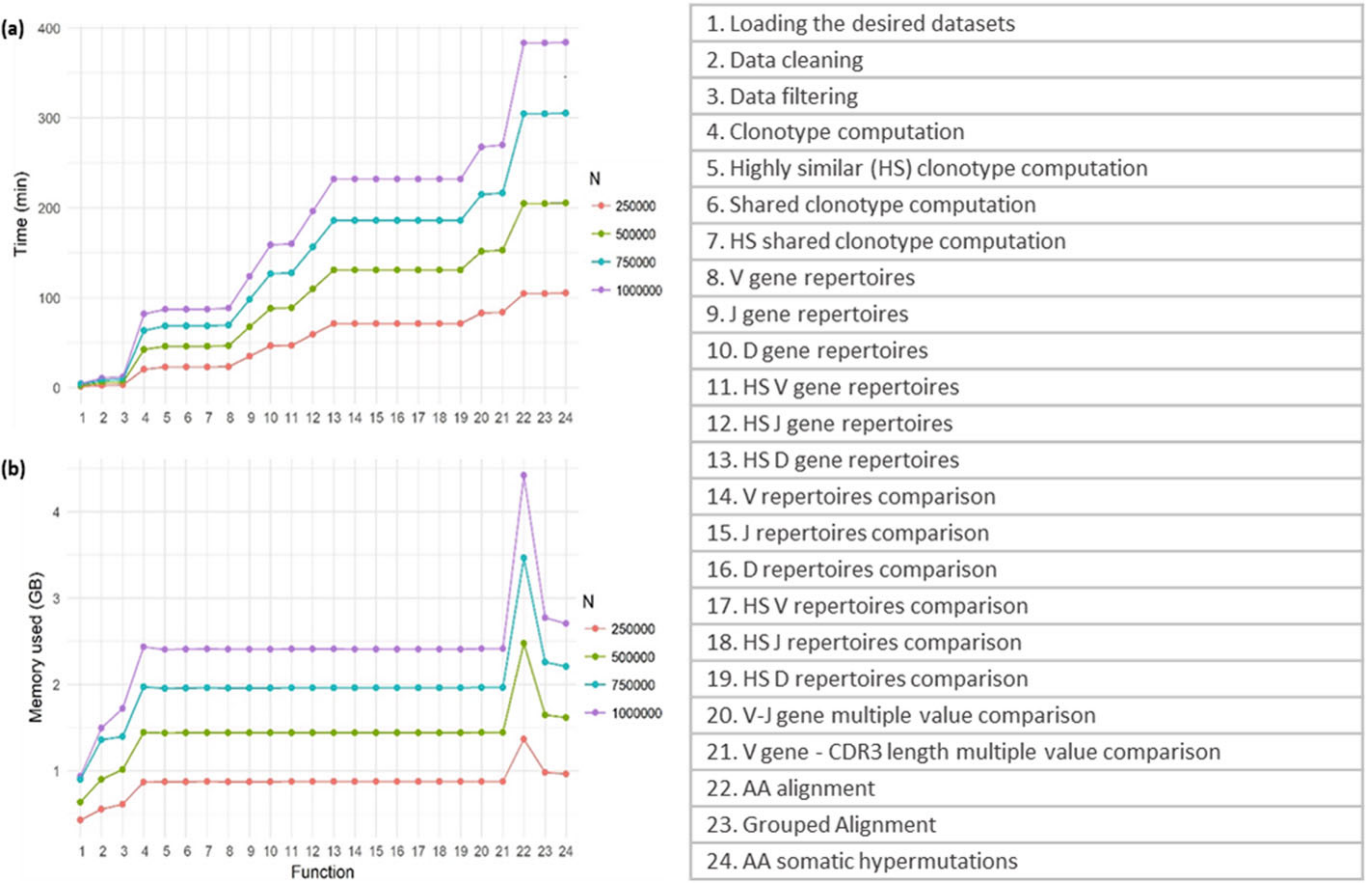
The computational time and the memory used (y-axis) of the graphical TRIP tool’s modules during a single pipeline run, for four different subsets of data. Each dataset’s size represents the total number of records for a particular sample (equivalent to the number of rows of the input tables), each dot on the time graph shows the elapsed time needed from the beginning of the pipeline until the corresponding procedure is terminated, and each dot on the memory graph represents the memory usage by the running routine. The functions which are used for the pipeline procedure are presented on the x-axis of both graphs

Figure 7 shows the memory usage and the time that has passed from the beginning of the process until the time when each module of the pipeline was completed. The total typical pipeline for BcR IG datasets of 1 million rows of data took approximately 6 h to run. The figure makes clear that the computational time and memory usage are increased linearly according to the dataset size.

Analyzing the time passed from one module to the next one, one can observe that the *V gene repertoires* and the *V gene Highly similar (HS) repertoires* processed are quicker than the corresponding *J* and *D repertoires*. The same thing for the aforementioned genes happens when they are involved in the *Multiple value comparison* process. The reason is that these processes are calculated based on the extracted clonotypes. In this pipeline, we computed the clonotypes using the *V gene* and the CDR3, so further computations are needed to find the major *J* and *D genes* of each group of clonotypes. The module of the pipeline with the highest memory and time demand is the *Alignment* process.

### Experimental results on a group of patients

The second set of experiments was focused on evaluating the efficiency of the TRIP tool. We assessed the reproducibility of the results produced by the main TRIP workflow with the results retrieved through the Galaxy IRProfiler [14]. As a control we used a previously published dataset [2] which consists of the TR gene rearrangements from three human samples. We checked the preselection and selection process, as well as the clonotype computation and the repertoire extraction modules. No significant differences were noted, whereas the clonotype and V gene repertoire were faithfully and reproducibly represented by TRIP. In more detail, although the preselection process seems to be stricter following the TRIP pipeline, almost 0.1% more rearrangements were filtered out, without having a significant impact on the final results. The clonotypes ranking and their relative frequencies remain comparable. (Tables 1, 2 and 3).

**Table 1:** Comparison of the results of the preselection and selection processes using the Galaxy and the TRIP tools

	**T3396 GALAXY**	**%**	**T3396 TRIP**	**%**	**T3397 GALAXY**	**%**	**T3397 TRIP**	**%**	**T3404 GALAXY**	**%**	**T3404 TRIP**	**%**
Total reads of raw data	348529	100	348529	100	372814	100	372814	100	296088	100	296088	100
Functional TRBV	340758	97.8	346629	99.5	367404	98.5	372014	99.8	289175	97.7	295277	99.7
filter out	1878	0.5	1900	0.5	796	0.2	800	0.2	772	0.3	811	0.3
Not Containing X,#,*	329344	94.5	335215	96.2	357235	95.8	361845	97.1	282510	95.4	288612	97.5
filter out	11414	3.3	11414	3.3	10169	2.7	10169	2.7	6665	2.3	6665	2.3
Productive	327600	94.0	326112	93.6	355740	95.4	354355	95.0	281129	94.9	279632	94.4
filter out	1744	0.5	9103	2.6	1495	0.4	7490	2.0	1381	0.5	8980	3.0
CDR3 landmarks C-F	325771	93.5	324289	93.0	354103	95.0	352725	94.6	279885	94.5	278396	94.0
filter out	1829	0.5	1823	0.5	1637	0.4	1630	0.4	1244	0.4	1236	0.4
Identity >= 95.0%	323109	92.7	321692	92.3	351084	94.2	349736	93.8	277287	93.7	275844	93.2
filter out	2662	0.8	2597	0.7	3019	0.8	2989	0.8	2598	0.9	2552	0.9
Total filter out	25420	7.3	26837	7.7	21730	5.8	23078	6.2	18801	6.3	20244	6.8
Total filter in	323109	92.7	321692	92.3	351084	94.2	349736	93.8	277287	93.7	275844	93.2
Number of Clonotypes	22743		22392		14359		14190		34304		33938	
Dominant Clonotype	TRBV4-1 CASSTTGTVDEKLFF		TRBV4-1 CASSTTGTVDEKLFF		TRBV4-1 CASSTTGTVDEKLFF		TRBV4-1 CASSTTGTVDEKLFF		TRBV28 CASSPPDTGELFF		TRBV28 CASSPPDTGELFF	
Dominant Clonotype Freq.		8.5		8.5093		14.8		14.8632		23.1		23.1

**Table 2:** Comparison of the results of the clonotype computation process using the Galaxy and the TRIP tools

**T3396 GALAXY**			**T3396 TRIP**		
**V-GENE**	**AA JUNCTION**	**Frequency %**	**V-GENE**	**AA JUNCTION**	**Frequency %**
TRBV4-1	CASSTTGTVDEKLFF	8.4826	TRBV4-1	CASSTTGTVDEKLFF	8.5093
TRBV12-3	CASSPNYSNQPQHF	3.5982	TRBV12-3	CASSPNYSNQPQHF	3.6485
TRBV5-4	CASSLGGTGELFF	2.4546	TRBV5-4	CASSLGGTGELFF	2.4594
TRBV20-1	CSARDGRDLEAQHF	1.9461	TRBV20-1	CSARDGRDLEAQHF	1.9503
TRBV19	CASSPRGFNTGELFF	1.3321	TRBV19	CASSPRGFNTGELFF	1.3341
TRBV4-1	CASSQRQGITGELFF	0.9452	TRBV4-1	CASSQRQGITGELFF	0.9471
TRBV11-1	CASSFWAGNTGELFF	0.7768	TRBV11-1	CASSFWAGNTGELFF	0.7780
TRBV27	CASSFTSAGELFF	0.7753	TRBV27	CASSFTSAGELFF	0.7737
TRBV29-1	CSVGGSGGTGYTF	0.6066	TRBV29-1	CSVGGSGGTGYTF	0.6064
TRBV27	CASRLGQAYGYTF	0.5531	TRBV27	CASRLGQAYGYTF	0.5483
**Top10 cumumative frequency**		21.4706			21.5567
**T3397 GALAXY**			**T3397 TRIP**		
**V-GENE**	**AA JUNCTION**	**Frequency %**	**V-GENE**	**AA JUNCTION**	**Frequency %**
TRBV4-1	CASSTTGTVDEKLFF	14.8369	TRBV4-1	CASSTTGTVDEKLFF	14.8632
TRBV19	CASSPRGFNTGELFF	6.4774	TRBV19	CASSPRGFNTGELFF	6.4848
TRBV12-3	CASSPNYSNQPQHF	4.1264	TRBV12-3	CASSPNYSNQPQHF	4.1631
TRBV5-1	CASSPSKGQGGTGELFF	2.1106	TRBV5-1	CASSPSKGQGGTGELFF	2.1095
TRBV13	CASSSDDSPLHF	2.0741	TRBV13	CASSSDDSPLHF	2.0747
TRBV4-1	CASSQRQGITGELFF	2.0391	TRBV4-1	CASSQRQGITGELFF	2.0449
TRBV12-5	CASGDTGAGNTIYF	1.7406	TRBV12-5	CASGDTGAGNTIYF	1.7404
TRBV20-1	CSARDGRDLEAQHF	1.6190	TRBV20-1	CSARDGRDLEAQHF	1.6226
TRBV10-2	CASSLDGMNYGYTF	1.3040	TRBV10-2	CASSLDGMNYGYTF	1.3026
TRBV7-6	CASSPRQGRNEKLFF	1.0923	TRBV7-6	CASSPRQGRNEKLFF	1.0942
**Top10 cumumative frequency**		37.4204			37.5006
**T3404 GALAXY**			**T3404 TRIP**		
**V-GENE**	**AA JUNCTION**	**Frequency %**	**V-GENE**	**AA JUNCTION**	**Frequency %**
TRBV28	CASSPPDTGELFF	23.0765	TRBV28	CASSPPDTGELFF	23.0543
TRBV7-2	CASRGGLYQPQHF	11.0932	TRBV7-2	CASRGGLYQPQHF	11.1012
TRBV29-1	CSVEDGQGPYSGNTIYF	2.1361	TRBV29-1	CSVEDGQGPYSGNTIYF	2.1345
TRBV18	CASSPTGGDSPLHF	0.9294	TRBV18	CASSPTGGDSPLHF	0.9273
TRBV15	CATSREGGEKLFF	0.7032	TRBV15	CATSREGGEKLFF	0.7040
TRBV5-1	CASSRSPLGTRDEQYF	0.6939	TRBV5-1	CASSRSPLGTRDEQYF	0.6935
TRBV15	CATSRDGQEYQPQHF	0.6661	TRBV15	CATSRDGQEYQPQHF	0.6641
TRBV29-1	CSVGGRLVGELFF	0.5738	TRBV29-1	CSVGGRLVGELFF	0.5746
TRBV15	CATSRDTLLAGEGGELFF	0.4378	TRBV15	CATSRDTLLAGEGGELFF	0.4372
TRBV29-1	CSVEYPGNGYTF	0.3101	TRBV29-1	CSVEYPGNGYTF	0.3099
**Top10 cumumative frequency**		40.6201			40.6008

**Table 3:** Comparison of the results of the repertoire extraction process using the Galaxy and the TRIP tools

**Vgene**	**T3396 GALAXY**	**T3396 TRIP**	**T3397 GALAXY**	**T3397 TRIP**	**T3404 GALAXY**	**T3404 TRIP**
TRBV10-1	0.2111	0.2144	0.1880	0.1903	0.0612	0.0619
TRBV10-2	0.5452	0.5493	1.0586	1.0712	0.2478	0.2475
TRBV10-3	1.4510	1.4648	0.9471	0.9443	3.4544	3.4799
TRBV11-1	1.9698	1.9873	2.0475	2.0437	0.3207	0.3241
TRBV11-2	2.8097	2.8448	2.1798	2.1987	2.2388	2.2571
TRBV11-3	0.3562	0.3617	0.0905	0.0916	0.1691	0.1709
TRBV12-3/12-4	10.2229	9.3114	8.1343	7.5476	9.2002	8.6687
TRBV12-5	0.9849	0.9914	1.9152	1.9309	0.8774	0.8840
TRBV13	0.6376	0.6476	2.7300	2.7484	0.1837	0.1856
TRBV14	0.4837	0.4912	0.4805	0.4863	0.6763	0.6836
TRBV15	0.7211	0.7324	0.6964	0.6977	1.6791	1.6943
TRBV18	3.9880	4.0461	2.9877	3.0233	2.9705	2.9996
TRBV19	7.6507	7.7662	7.5423	7.6321	8.0340	8.1089
TRBV2	1.8071	1.8265	1.9779	1.9944	0.7055	0.7131
TRBV20-1	4.0672	4.1220	4.0811	4.1297	3.0929	3.1263
TRBV24-1	0.3386	0.3439	0.2229	0.2255	1.2127	1.2199
TRBV25-1	0.4749	0.4778	0.4805	0.4863	0.3673	0.3713
TRBV27	5.5358	5.5913	6.5743	6.6173	4.4368	4.4729
TRBV28	2.1897	2.2061	2.8066	2.8330	2.6207	2.6372
TRBV29-1	8.1036	8.1904	8.2736	8.3298	7.3024	7.3664
TRBV30	0.2506	0.2546	0.0209	0.0211	3.1600	3.1941
TRBV3-1	0.2023	0.2054	0.1045	0.1057	0.0262	0.0265
TRBV4-1	2.6953	2.7331	3.1409	3.1783	0.6734	0.6777
TRBV4-2	0.6024	0.6118	0.2995	0.3030	1.9764	1.9978
TRBV4-3	0.0396	0.0402	0.0279	0.0282	1.5683	1.5852
TRBV5-1	7.8750	7.9895	7.6746	7.7590	8.4276	8.4978
TRBV5-4	5.0169	5.0956	4.2343	4.2777	3.9820	4.0191
TRBV5-5	2.4667	2.3669	2.0475	1.9662	3.1017	3.0320
TRBV5-6	3.1878	3.2199	2.9529	2.9528	3.6293	3.6537
TRBV5-8	0.2594	0.2635	0.5223	0.5285	0.1603	0.1621
TRBV6-1	2.4579	2.4964	2.6395	2.6709	2.4691	2.4957
TRBV6-2	1.9435	1.9561	2.6743	2.6850	2.7752	2.7756
TRBV6-4	0.7871	0.7994	0.1671	0.1691	0.9299	0.9399
TRBV6-5	6.3272	6.3326	6.6160	6.5610	5.5767	5.5071
TRBV6-6	2.1897	2.2106	2.1659	2.1776	2.7548	2.7756
TRBV6-8	0.0484	0.0491	0.0000	0.0000	0.0087	0.0088
TRBV6-9	0.0088	0.0089	0.0766	0.0775	0.0408	0.0413
TRBV7-2	2.2908	2.3223	1.7062	1.7266	5.9993	6.0198
TRBV7-3	0.8090	0.8173	0.5571	0.5638	0.1079	0.1090
TRBV7-4	0.0835	0.0804	0.0975	0.0987	0.0175	0.0177
TRBV7-6	0.3386	0.3349	0.8496	0.8598	0.1632	0.1650
TRBV7-7	0.1187	0.1206	0.0696	0.0705	0.0321	0.0324
TRBV7-8	2.1589	2.1793	3.0295	3.0585	0.9999	1.0107
TRBV7-9	2.9943	3.0413	2.6255	2.6568	1.4313	1.4468
TRBV9	0.2990	0.3037	0.2855	0.2819	0.1370	0.1355

### Comparison to the galaxy IRProfiler tool

We used IRProfiler - a software toolbox for high throughput immune receptor profiling [14], which is available through the Galaxy platform, in order to compare its functionalities with the TRIP tool. The main differences are listed in Table 4.

**Table 4:** Comparison of functionalities offered by TRIP and the Galaxy IRProfiler tools

**Feature**	**TRIP**	**GALAXY IRProfiler**
Data processing	Multiple samples processing per session	One sample per session
Data filtering	Two stages of filtering: preselection and selection with filtering choices for V-Region, CDR3 and V-D-J gene	One step of data filtering with same parameters. Indels are also included
Clonotype computation	There are 10 different clonotype definitions from which the user might choose. Convergent evolution of each clonotype is also computed, when possible. Linking each clonotype with the sequences (and all related information) which are assigned to, is also possible.	Only three clonotype definitions: V+CDR3, J+CDR3, CDR3
Highly similar computation	Highly similar clonotypes merged based on user defined CDR3 thresholds	Not supported
Repertoires extraction	Multiple repertoire extraction for V-D-J gene (and allele). Choice of repertoire extraction based on highly similar clonotypes given	Only V and J gene repertoire extraction
Repertoire comparison	Comparison of V, D and J gene and allele usage among multiple repertoires	Comparison of gene usage for V and J subgroups among multiple repertoires
Shared clonotypes	Shared clonotypes among datasets including/excluding singletons, V-gene (based on the clonotypes definition)	Shared clonotypes among datasets including/excluding singletons. In order to exclude the V gene users have to re-analyze the datasets
CDR3 distribution	CDR3 distribution with output visualization plots	Not supported
Pi distribution	Pi distribution with output visualization plots	Not supported
Multiple value comparison	Multiple comparisons between V-D-J gene, molecular mass, and pI	Not supported
Alignment	An alignment table is computed for the selected region (V-D-J REGION, V-J REGION). A grouped alignment table is computed as well. The selected region can be aligned at nucleotide level, at amino acid level or both. The reference sequences used can be at allele level or at gene level. The user can also insert his/her own reference sequence.	Not supported
Somatic hypermutations	A table with the mutations based on alignment table is computed	Not supported
Visualization	Output bar plots, pie charts and logo graphs is supported	Not supported

## Conclusions

NGS holds the potential to offer new knowledge of both biological and clinical relevance for improved understanding of: (i) many normal processes and mechanisms, such as B and T cell development, inflammation and the aging of the immune system, and (ii) pathological conditions, such as cancer and autoimmunity.

However, the interpretation of the results and the extraction of meaningful conclusion requires extensive expertise in bioinformatics, which is often limited in clinical as well as science laboratories. To this end, capitalizing on our long-standing experience into the field of immunogenetics and bioinformatics analysis in the field, we designed a user-friendly, straightforward bioinformatics pipeline in order to assist not only the inexperienced users but also the experienced ones by facilitating the analytical part.

TRIP is a novel software framework that provides analytics services on antigen receptor gene sequence data, offering the opportunity for an in-depth analysis based on the processing of the output files of the IMGT/HighV-Quest tool. It provides detailed information about V, D and J gene usage, CDR3 AA and Nt composition, and clonality, also offering analysis of the SHMs present in the V-region of the BcR IGs. It is accurate, open source, easy-to-use, user friendly, and enables the user to build a personalized pipeline. Finally, it processes many different datasets at the same time.

TRIP can be utilized to characterize the enormous complexity of the immune repertoire of a given case in terms of clonal composition and repertoire analysis. It also offers the opportunity to study the intraclonal temporal dynamics, i.e. clonal drift, and the subclonal ’architecture’ of the BcR IG gene repertoire, essentially arising from intraclonal diversification of the IG genes in the context of ongoing SHM that may lead to extensive ’branching’ of the clone. Moreover, TRIP can be used for the determination of IGHV gene SHM status and to monitor clonal expansions.

## Availability and requirements

- **Project name:** TRIP
- **Project homepage:**https://bio.tools/TRIP_-_T-cell_Receptor_Immunoglobulin_Profiler
- **Operating system:** Platform independent
- **Programming language:** R
- **Other requirements:** See GitHub page
- **License:** GNU GPL
- **Any restrictions to use by non-academics:** None

## Data Availability

The IMGT High-VQuest output files that were used as input to TRIP for the scalability experiments, are available on FigShare here 10.6084/m9.figshare.11881713 - the file IDs are BC23-OSR052411, BC23-OSR052411-OSR081811, OSR052311-OSR081811 and OSR052411-OSR052311-OSR081811. The corresponding raw FASTQ files are available here: https://www.ebi.ac.uk/ena/browser/view/PRJEB29674. The IMGT High-VQuest output files that were used as input to Galaxy and TRIP for the comparison, are available on FigShare here 10.6084/m9.figshare.11881713- the file IDs are T3304, T3396 and T3397. Raw TR sequence data can be found under accession number SRR3737053 in GenBank sequence database www.ncbi.nlm.nih.gov/genbank/.

## Abbreviations

AA: Aminoacid
NT: Nucleotide
BcR: B-cell receptor
TcR: T-cell receptor
CDR3: Complementarity-determining region 3
IG: Immunoglobulin
IMGT: International ImMunoGeneTics information system
NGS: Next generation sequencing

## References

[1] Rawstron A, Fazi C, Agathangelidis A, Villamor N, Letestu R, Nomdedeu J, Palacio C, Stehlikova O, Kreuzer K, Liptrot S (2016). A complementary role of multiparameter flow cytometry and high-throughput sequencing for minimal residual disease detection in chronic lymphocytic leukemia: an european research initiative on cll study. Leukemia.

[2] Vardi A, Vlachonikola E, Karypidou M, Stalika E, Bikos V, Gemenetzi K, Maramis C, Siorenta A, Anagnostopoulos A, Pospisilova S (2017). Restrictions in the t-cell repertoire of chronic lymphocytic leukemia: high-throughput immunoprofiling supports selection by shared antigenic elements. Leukemia.

[3] Rodríguez-Vicente AE, Bikos V, Hernández-Sánchez M, Malcikova J, Hernández-Rivas J-M, Pospisilova S (2017). Next-generation sequencing in chronic lymphocytic leukemia: recent findings and new horizons. Oncotarget.

[4] Thomas N, Heather J, Ndifon W, Shawe-Taylor J, Chain B (2013). Decombinator: a tool for fast, efficient gene assignment in t-cell receptor sequences using a finite state machine. Bioinformatics.

[5] Bolotin DA, Shugay M, Mamedov IZ, Putintseva EV, Turchaninova MA, Zvyagin IV, Britanova OV, Chudakov DM (2013). Mitcr: software for t-cell receptor sequencing data analysis. Nat Methods.

[6] Yang X, Liu D, Lv N, Zhao F, Liu F, Zou J, Chen Y, Xiao X, Wu J, Liu P (2015). Tcrklass: a new k-string–based algorithm for human and mouse tcr repertoire characterization. J Immunol.

[7] Kuchenbecker L, Nienen M, Hecht J, Neumann AU, Babel N, Reinert K, Robinson PN (2015). Imseq—a fast and error aware approach to immunogenetic sequence analysis. Bioinformatics.

[8] Lefranc M-P, Giudicelli V, Duroux P, Jabado-Michaloud J, Folch G, Aouinti S, Carillon E, Duvergey H, Houles A, Paysan-Lafosse T (2014). Imgt®, the international immunogenetics information system® 25 years on. Nucleic Acids Res.

[9] Alamyar E, Duroux P, Lefranc M-P, Giudicelli V (2012). Imgt® tools for the nucleotide analysis of immunoglobulin (ig) and t cell receptor (tr) v-(d)-j repertoires, polymorphisms, and ig mutations: Imgt/v-quest and imgt/highv-quest for ngs. Immunogenetics.

[10] Aouinti S, Malouche D, Giudicelli V, Kossida S, Lefranc M-P (2015). Imgt/highv-quest statistical significance of imgt clonotype (aa) diversity per gene for standardized comparisons of next generation sequencing immunoprofiles of immunoglobulins and t cell receptors. PLoS ONE.

[11] Aouinti S, Giudicelli V, Duroux P, Malouche D, Kossida S, Lefranc M-P (2016). Imgt/statclonotype for pairwise evaluation and visualization of ngs ig and tr imgt clonotype (aa) diversity or expression from imgt/highv-quest. Front Immunol.

[12] Bolotin DA, Poslavsky S, Mitrophanov I, Shugay M, Mamedov IZ, Putintseva EV, Chudakov DM (2015). Mixcr: software for comprehensive adaptive immunity profiling. Nat Methods.

[13] Duez M, Giraud M, Herbert R, Rocher T, Salson M, Thonier F (2016). Vidjil: a web platform for analysis of high-throughput repertoire sequencing. PLoS ONE.

[14] Maramis C, Gkoufas A, Vardi A, Stalika E, Stamatopoulos K, Hatzidimitriou A, Maglaveras N, Chouvarda I (2018). Irprofiler–a software toolbox for high throughput immune receptor profiling. BMC Bioinformatics.

[15] Pommié C, Levadoux S, Sabatier R, Lefranc G, Lefranc M-P (2004). Imgt standardized criteria for statistical analysis of immunoglobulin v-region amino acid properties. J Mol Recog.

[16] Vardi A, Vlachonikola E, Papazoglou D, Psomopoulos F, Kotta K, Ioannou N, Galigalidou C, Gemenetzi K, Pasentsis K, Kotouza M, Koravou E, Scarfò L, Iskas M, Stavroyianni N, Ghia P, Anagnostopoulos A, Kouvatsi A, Ramsay AG, Stamatopoulos K, Chatzidimitriou A. T cell dynamics in chronic lymphocytic leukemia under different treatment modalities. Clin Cancer Res. 2020. https://doi.org/10.1158/1078-0432.CCR-19-3827. https://clincancerres.aacrjournals.org/content/early/2020/07/02/1078-0432.CCR-19-3827.full.pdf. 10.1158/1078-0432.CCR-19-3827 32616500

[17] Vardi A, Vlachonikola E, Mourati S, et al. High-throughput b-cell immunoprofiling at diagnosis and relapse offers further evidence of functional selection throughout the natural history of chronic lymphocytic leukemia. HemaSphere. 2019; 3(512). 10.1097/01.hs9.0000562808.48237.52.

[18] Vlachonikola E, Vardi A, Kastritis E, et al. Longitudinal t cell immunoprofiling of patients with relapsed and/or refractory myeloma who receive daratumumab monotherapy: A subanalysis of a phase 2 study (the rebuild study). Blood. 2019; 134(Supplement13167). 10.1182/blood-2019-124655.

[19] Gemenetzi K, Stalika E, Agathangelidis A (2018). Evidence for epitope-specific t cell responses in hiv-associated non neoplastic lymphadenopathy: High-throughput immunogenetic evidence. Blood.

[20] Gemenetzi K, Agathangelidis A, Sutton L-A (2018). Remarkable functional constraints on the antigen receptors of cll stereotyped subset 2: High-throughput immunogenetic evidence. Blood.

[21] Galigalidou C, Papadopoulou A, Stalika E (2018). High-throughput t cell receptor (tr) repertoire analysis of virus-specific t cells: Implications for t cell immunotherapy and viral infection risk stratification. Blood.

[22] Venturi V, Kedzierska K, Price DA, Doherty PC, Douek DC, Turner SJ, Davenport MP (2006). Sharing of t cell receptors in antigen-specific responses is driven by convergent recombination. Proc Natl Acad Sci.

[23] Madi A, Shifrut E, Reich-Zeliger S, Gal H, Best K, Ndifon W, Chain B, Cohen IR, Friedman N (2014). T-cell receptor repertoires share a restricted set of public and abundant cdr3 sequences that are associated with self-related immunity. Genome Res.

[24] Venturi V, Price DA, Douek DC, Davenport MP (2008). The molecular basis for public t-cell responses?. Nat Rev Immunol.

[25] Roy A, Bystry V, Bohn G, Goudevenou K, Reigl T, Papaioannou M, Krejci A, O’Byrne S, Chaidos A, Grioni A (2017). High resolution igh repertoire analysis reveals fetal liver as the likely origin of life-long, innate b lymphopoiesis in humans. Clin Immunol.

[26] Merkel D (2014). Docker: lightweight linux containers for consistent development and deployment. Linux J.

[27] IMGT/HighV-Quest Tool. https://www.imgt.org/HighV-QUEST/login.action. Accessed 6 Aug 2020.

